# Cache-Based Privacy Protection Scheme for Continuous Location Query

**DOI:** 10.3390/e25020201

**Published:** 2023-01-19

**Authors:** Zhenpeng Liu, Dewei Miao, Ruilin Li, Yi Liu, Xiaofei Li

**Affiliations:** 1 Information Technology Center, Hebei University, Baoding 071002, China; 2 School of Cyber Security and Computer, Hebei University, Baoding 071002, China

**Keywords:** variable-order Markov model, location caching, differential privacy, *k*-anonymity, location protection

## Abstract

Users who initiate continuous location queries are prone to trajectory information leakage, and the obtained query information is not effectively utilized. To address these problems, we propose a continuous location query protection scheme based on caching and an adaptive variable-order Markov model. When a user initiates a query request, we first query the cache information to obtain the required data. When the local cache cannot satisfy the user’s demand, we use a variable-order Markov model to predict the user’s future query location and generate a *k*-anonymous set based on the predicted location and cache contribution. We perturb the location set using differential privacy, then send the perturbed location set to the location service provider to obtain the service. We cache the query results returned by the service provider to the local device and update the local cache results according to time. By comparing the experiment with other schemes, the proposed scheme in this paper reduces the number of interactions with location providers, improves the local cache hit rate, and effectively ensures the security of the users’ location privacy.

## 1. Introduction

With the rapid development of Internet technology, GPS technology and mobile devices, location-based services (LBS) have been widely used in people’s lives [1,2], such as mobile navigation and point-of-interest queries. Users obtain the needed information by sending their location information to the location service providers (LSPs). However, while enjoying the convenience of location services, malicious LSPs may leak a users’ location information [3]. The users’ sensitive information, such as their home and work addresses, identity information, and the user’s life path, can be inferred by analyzing location data [4,5]. When the users’ sensitive information is leaked, users may suffer significant losses. Therefore, location privacy protection is a hot issue in user privacy protection research.

There is a large amount of research on the issue of location privacy protection, and the techniques are broadly classified as fuzzy, encrypted, anonymous, and differential privacy. Fuzzy-based technology prevents attackers from directly obtaining user location information by adding perturbations to the user’s location [6,7]. Encryption-based technology usually encrypts the location query request as a whole, which can ensure user privacy and service quality, but the communication process and calculation overhead are high [8]. Anonymous technology uses the generated anonymity set to initiate queries by forming an anonymous region with other user locations, and the common method is mainly *k*-anonymity technology. Anonymous techniques are vulnerable to attackers using known background knowledge to launch replay attacks and are prone to waste resources. The differential privacy technique is based on strict mathematical theory. It perturbs the location set by adding noise to ensure that the location set is not affected by a particular data change and prevents attackers from using background knowledge to obtain user data. The commonly used methods are the Laplace mechanism and the exponential mechanism. In addition to differential privacy, some scholars have used secure multi-party computing to protect users’ location privacy in recent work [9]. Secure multi-party computation enables multiple participants holding their respective private data to perform a series of operations and obtain computational results based on cryptographic theory without revealing their respective actual data. Unlike differential privacy, secure multi-party computation provides a higher degree of privacy protection during computation; however, it does not provide better protection for computation results and has high computation and communication overhead. In addition, there are applications of caching techniques in location protection to store location information locally to avoid untrustworthy LSPs from leaking user-location privacy. The current main research direction is to improve the local cache hit rate [10,11]. The cache hit ratio refers to the ratio of the number of queries fetched from the local cache to the total number of queries. The higher the hit ratio, the fewer the number of queries initiated by users to the LSP, the higher the privacy of users, and the lower the system overhead.

LBS privacy protection architecture is mainly divided into centralized and distributed architecture [12,13], the centralized architecture contains an anonymous server between users and LSP, and the location transmission service is performed through the server. However, the reliability of third-party servers is usually not guaranteed in a centralized architecture and can easily become a bottleneck for system security. Distributed architecture in which the user initiates a request for information directly to the LSP avoids information leakage from an unreliable third-party.

To address the problems of the existing methods, we propose a cache-based privacy protection scheme for continuous location service. A distributed architecture is used to avoid privacy leaks by untrustworthy third parties while avoiding communication overhead with middle servers. The main contributions of this paper are as follows:

1. A new location caching scheme is proposed that uses caching techniques to reduce the number of interactions with LSPs, improves the cache hit rate through the designed query scheme and reduces the risk of privacy leakage in continuous location queries.
2. When it is necessary to initiate a query to the LSPs, we use differential privacy techniques to perturb the anonymous location set to ensure the user’s location privacy during the query.
3. By comparing with other schemes in terms of cache hit rate, query time, and degree of privacy protection, our scheme can better protect user-location privacy and reduce the overhead of communication with LSP.

The remainder of the article is as follows. We present the related work in Section 2. Section 3 introduces the system model and related definitions in this paper. Section 4 details the scheme of this paper, and the security analysis of the proposed scheme is presented in Section 5. Section 6 presents the comparative tests of this paper with other schemes and analyzes them. Finally, conclusions are drawn.

## 2. Related Work

### 2.1. k-Anonymity Technology

In location privacy protection research, anonymity-based *k*-anonymity techniques are widely used, which were first proposed by Sweeney [14]; this technology uses attribute generalization to make one data record indistinguishable from other *k* − 1 data records. Gruteser M et al. [15] first used the *k*-anonymity technique as a means of location privacy protection by constructing a *k*-anonymity location model through quadtree search to ensure that the anonymity region is not less than a certain value. However, this method increases the time overhead and is prone to anonymous location overload. Ling et al. [16] constructed a distributed location privacy protection mechanism based on offset grids to solve the problem of untrustworthy anonymous servers. Zhang et al. [17] combined the idea of *k*-anonymity to generate anonymous polygonal regions using an irregular polygon generation algorithm.

### 2.2. Caching Technology

Caching technology reduces the number of user interactions with LSPs by caching the location data queried at LSPs locally, receiving wide application in location privacy protection. Zhu et al. [18] designed the mobile-cache system by adding a location caching mechanism to *k*-anonymity, which improves the cache hit rate by selecting locations that have not been queried before when constructing anonymity sets and reduces the resource overhead and resource utilization by reducing the number of queries. Chen et al. [10] proposed a location protection scheme for distributed caching. The cache information is stored in the distributed cache layer, and Markov chains are used to construct query sets, which improve the cache hit rate. However, the distributed cache layer reduces the query efficiency. The scheme uses *k*-anonymity sets to initiate queries to LSPs without considering the background knowledge of locations in the anonymity set. Jung et al. [19] proposed a location protection scheme based on P2P architecture, which protects user-location privacy in LBS by storing data locally and introduces a collaborative caching technique to improve performance by sharing query results among users. Hu et al. [20] constructed *k*-anonymous sets in continuous location queries to query multiple locations simultaneously and cache the results locally. However, they did not consider background knowledge attacks. Zhang et al. [21] used a grid structure to cache data and encrypt the sent query requests using symmetric encryption and *k*-anonymity techniques. However, the security of the key cannot be guaranteed in continuous queries. Zhang et al. [22] used a multi-level caching approach in continuous LBS to add query caches in local and third-party servers, but it has the risk of server leakage of privacy. To avoid problems with third parties, Cui et al. [23] queried other users to obtain services from neighboring devices. However, malicious neighboring points can compromise user privacy. Zhu et al. [11] used a variable-order Markov model to predict the user’s future location when initiating a query, combined with the probability of predicted locations to randomly select virtual locations to construct a *k*-anonymity set, and saved the query results in a central server. However, this method does not consider the zero-frequency problem in the model. In other words, there is no historical track matching the current path, nor does the number of predicted locations meet the *k*-anonymity requirement. Nisha et al. [24] shared local caches by creating one-time spatial groups with neighbors and protected personal privacy by generating virtual locations and identities. The security of neighbors was also not guaranteed. Huang et al. [25] applied caching techniques to vehicle trajectory privacy protection by caching the location services returned by LSPs in a roadside unit in the vehicle system architecture and using virtual locations to initiate service requests to LSPs.

### 2.3. Differential Privacy

Differential privacy (DP) was first proposed by Dwork [26] in 2006. Wang et al. [27] proposed a location protection method based on differential privacy perturbation, which sends the perturbed location information to the service provider to achieve location protection. Zhang et al. [28] used a center-clustering algorithm based on the max–min distance to generate multiple sets of candidate virtual objects and select the optimal virtual candidate set to achieve *k*-anonymity. Zhang et al. [29] protected users’ location privacy through the Laplace mechanism and used the indexing mechanism to protect the query privacy of users. Li et al. [30] used the Markov model to predict the user location and added Laplace noise to the two nodes with the largest predicted values to protect user location. Zhang et al. [31] used differential privacy to protect user trajectory data. They balanced privacy and utility by assigning privacy levels to location points in trajectories through semantic analysis and assigning corresponding privacy budgets according to the privacy levels. Kou et al. [32] designed a location privacy-preserving algorithm and proposed a privacy-preserving scheme for location in sensor networks based on differential privacy, which ensures the utility of the data while adding noise.

The existing research on cache-based location protection has a low local cache hit rate, and the location information is easily leaked when users initiate queries to LSPs. In order to address the problems in previous studies, this paper combines *k*-anonymity and variable-order Markov prediction models to design a continuous location privacy-protection scheme based on caching technology and without TTP. We propose an anonymity set generation algorithm based on the variable-order Markov model and location cache contribution, which solves the problem of too few prediction results of the Markov model and improves the local cache hit rate. Using differential privacy techniques to perturb the anonymity set improves the location privacy security when users initiate queries.

## 3. System Model and Definition

This section first clarifies the objectives and use scenarios of the system, then introduces the system model of this paper; after that, it defines the attack model, and finally introduces the variable-order Markov model and differential privacy techniques.

### 3.1. Objectives and Use Scenarios

When users need location-related services, they need to initiate location-related query requests to LSPs and return data through LSPs to meet their needs. However, there is also a risk of leaking user privacy in this process. We propose a scenario in which users keep sending their location information and query requests to LSPs while they are on the move. There are two problems: firstly, during the process of users continuously initiating queries, untrustworthy LSPs can obtain users’ mobile trajectories through the requests sent by users and thus infer users’ privacy; secondly, many duplicate queries will be generated, which results in a great deal of wasted resources and increases the risk of location leakage.

We designed a cache-based continuous location privacy protection scheme to address the above problems. For the first problem, we used the *k*-anonymity technique to obfuscate user locations. To prevent attackers from using background knowledge attacks to obtain user locations, we used differential privacy techniques to perturb on top of *k*-anonymity to ensure users’ privacy during the query process. For the second problem, we used caching techniques to cache the query results locally and update the local cache information by LSP return information and cache threshold to ensure the availability of cached data.

### 3.2. System Model

**Figure 1:**
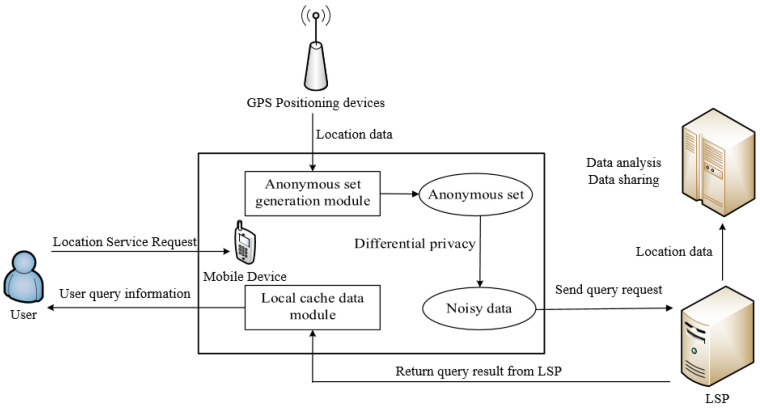
System framework model.

Our scheme adopts a distributed architecture consisting mainly of two parts: local users and LSPs, as shown in Figure 1. After users obtain their own location information through wireless devices, they first query whether the required information exists in the local cache. If it exists, they directly obtain the data without initiating a query. When the required service is unavailable in the cache, a *k*-anonymous location set containing the user’s current location and other *k* − 1 locations is constructed by the proposed privacy-preserving scheme. Our scheme only considers location protection for a single user, and to reduce computation and communication overhead, we use differential privacy techniques to perturb the generated *k*-anonymous location set to prevent background knowledge attacks. The location set is sent to the LSP to obtain location services, and the LSP provides query results back to the local user, presents the required information to the user based on the query results, and updates the local cache data.

### 3.3. Attack Model

When users use *k*-anonymity techniques in LBS services, attackers mainly use background knowledge attacks, probabilistic attacks, and semantic attacks to obtain the user location. Background knowledge attack means that the attacker obtains the location information with high probability based on the background knowledge information they have. Probabilistic attack means the attacker filters out unreasonable location points through available information, thereby increasing the probability of discovering the user’s actual location. There are many forms of semantic attacks, among which the positional homogeneity attack is a common attack in semantic attacks. A homogeneity attack refers to the attacker finding the user’s location by narrowing the scope.

In the process of users initiating location queries, assuming that the attackers have the privacy protection mechanism used by the user, they can use the above-mentioned attack methods to launch attacks on the user’s location and identity. The attackers are mainly data eavesdroppers in the process of obtaining location services and malicious LSPs.

### 3.4. Variable-Order Markov Model

The Markov model can predict the possible location of the user in the future, and the cache hit rate can be improved by querying the predicted location [33].

For an *m*-order Markov chain, the user’s next moment of occurrence location is determined by the previous *m* locations.

(1) $$Pr(L_{n+1}=l\;|\;L_{n}=l_{n},L_{n-1}=l_{n-1},\cdots ,L_{1}=l_{1})=Pr(L_{n+1}=l\;|\;L_{n}=l_{n},L_{n-1}\;=l_{n-1},\cdots ,L_{n-m+1}=L_{n-m+1})\;(n>m)$$

The probability of occurrence of $\left(L_{n-m+1},\cdots ,L_{n}\right)$ in the statistical sample is as follows:

(2) $$Pr(L_{n-m+1},\cdots ,L_{n})\;=Pr(L_{n-m+1},\cdots ,L_{n})Pr(L_{n-m+2}\;|\;L_{n-m+1})\cdots Pr(L_{n}\;|L_{n-m+1},\cdots ,L_{n-1})$$

We constructed the first-order to *m*-order model and used the longest matching principle to predict with the highest-order model. From Equation (2), we know that when the trajectory length does not satisfy the order, the next position cannot be predicted, so the prediction can be made by using the descending order until it is reduced to the first order. In this way, the flexibility of the variable-order Markov model can be demonstrated, and the prediction efficiency can be guaranteed.

### 3.5. Differential Privacy

Differential privacy techniques can protect user location well by adding noise to the data in the dataset and do not reduce the availability of location data. The relevant location differential privacy is defined as follows.

**Definition 1.**  ε-location differential privacy [27]. There exist n positions, each corresponding to one record. Given a privacy algorithm N and its definition domain Def(N) and value domain Ran(N), if any two neighboring position sets t and t′ (t, t′∈Def(N)) satisfy the same output result t* (t*∈Ran(N)) and satisfy Equation (3), it is shown that algorithm N satisfies ε-location differential privacy.

(3) $$Pr\left(N\left(t\right)=t^{*}\right)\leq e^{\varepsilon }Pr\left(N\left(t^{\prime }\right)=t^{*}\right)$$

**Definition 2.**  *Global sensitivity [27,30]. Global sensitivity is an important metric for differential privacy-preserving algorithms. For any function f: D → R^d^, the global sensitivity of f is defined as:*

(4) $$\Delta f=\underset{D,D^{\prime }}{\max}{\left|\left|f\left(D\right)-f\left(D^{\prime }\right)\right|\right|}_{1}$$

*where D and D′ denote adjacent datasets where there is at most one different piece of information, and* ${\left|\left|f\left(D\right)-f\left(D^{\prime }\right)\right|\right|}_{1}$ *is the first-order norm value between f(D) and f(D′).*

*The commonly used differential privacy protection mechanisms are the Laplace mechanism and exponential mechanism, among which the Laplace mechanism is mainly used for numerical data, and the exponential mechanism is generally used for non-numerical data. In this paper, the Laplace mechanism is used to add noise.*

**Definition 3.**  *Laplace mechanism [30]. Given a location dataset D, for any function f: D → R^d^ with sensitivity Δf, if the output of function f satisfies Equation (5):*

(5) $$M\left(D\right)=f\left(D\right)+Lap\left(\frac{\Delta f}{\varepsilon }\right)$$

*We say that the function f satisfies ε-differential privacy, where* $Lap\left(\frac{\Delta f}{\varepsilon }\right)$ *is random noise with the magnitude of the noise amount proportional to the global sensitivity Δf and inversely proportional to the privacy budget ε.*

## 4. Cache-Based Location Privacy Protection Scheme

This section mainly introduces the proposed location protection scheme. When initiating a query, firstly, a variable-order Markov model is used to predict the possible future locations of the user and generate a *k*-anonymous set with the current location. When the predicted locations do not satisfy *k*-anonymity, the location cache contribution in a range of the user’s region is considered, and the location points with large cache contributions are selected to complete the *k*-anonymity set. Finally, Laplacian noise is added to the generated anonymity set to prevent LSPs from obtaining user locations directly. The query results are used to update the local cache data and reduce the number of interactions with LSPs. We describe the storage form of location data in the local cache in Section 4.1, and then in Section 4.2, we introduce the generation method of the anonymous set required when launching queries to LSPs in the scheme. Table 1 lists the important symbols used in our scheme for reference.

### 4.1. The Structure of the Local Cache Data

The location data acquired by mobile devices are usually geographic coordinate values, usually divided into location units when performing data analysis. In this paper, the scheme proposed by Pinelli [34] is used to grid the geographic location. Firstly, the user activity area is divided into a rectangular area, then the area is divided into *M* × *N* square grids of size 10 m × 10 m, and finally, the coordinate data are mapped into the grid, and the grid sequence is used to store the location information. The current user coordinates are (*p_i_*, *q_i_*), then the grid area mapped to the grid is as follows.

(6) $$\left(x_{i},y_{i}\right)=\lceil \frac{\left(p_{i}-p_{d}\right)M}{p_{t}-p_{d}},\frac{\left(q_{i}-q_{d}\right)N}{q_{t}-q_{d}}\rceil$$

where (*p_t_*, *q_t_*) and (*p_d_*, *q_d_*) are the coordinates of the top-right and bottom-left vertices of the rectangular region, respectively. Each cached data is of the form *Q* = {(*x_i_*, *y_i_*), *data*, *t*}, *t* is decreasing with time, and the location data is removed from the local cache when the availability period is exceeded, and the available time is refreshed when the cached data is updated.

### 4.2. Anonymous Set Generation

When the user cache information cannot satisfy the user query demand, the query service needs to be initiated to the LSP. Our scheme combines variable-order Markov models and cache contributions to generate *k*-anonymous sets. A variable-order Markov model from first-order to *m*-order is constructed from the user’s historical dwell points, and the largest order model available is automatically selected for location prediction based on the length of the user’s historical trajectory.

#### 4.2.1. Obtain Stay Points

The stay point refers to a location point where the user stays for a certain time within a certain distance. Users usually stay at a stay point for a certain amount of time to complete something and hardly move during this time, so the relationship between location points can be judged based on the speed limit. The user history trajectory is denoted as $L=\left\{l_{1},l_{2},\cdots ,l_{n}\right\}$, where $l_{n}=\{(x_{n},y_{n}),t_{n}\}$ denotes the geographic coordinates where the user is located at time *t_n_*. The relationship between *a* and *b* is shown in Equation (7).

(7) Rea,b=expdista,bta−tb−δ

where δ is the set speed threshold and dist(*a,b*) denotes the distance between *a* and *b*.

(8) $$\mathrm{dist}\left(a,b\right)=R_{earth}*\arccos(\sin\left(X_{a}\right)\sin\left(X_{b}\right)+\cos\left(X_{a}\right)\cos\left(X_{b}\right)\cos\left(Y_{a}-Y_{b}\right))$$

where $X_{i}$ and $Y_{i}$ are the units of radians of latitude and longitude. $R_{earth}$ is the radius of the earth, which is given in meters. We can define a set of location points $M=\left(l_{m},l_{m+1},\cdots ,l_{n}\right)$ as stay points, which satisfy Rela,lb≤1 m≤a,b≤n, and use the center point of *M* to denote the coordinates of each set of stay points, expressed as Equation (9).

(9) $$c=\left(\frac{\sum_{j=m}^{n}x_{j}}{\left|l\right|},\frac{\sum_{j=m}^{n}y_{j}}{\left|l\right|},\frac{t_{r}-t_{o}}{2}\right)$$

where $\left|l\right|$ is the number of location points in *M*, $t_{o}$ and $t_{r}$ denoting the start time and end time of the location points. The stay point coordinates are mapped to the divided grid region, and the set of the user moving location regions $P=\left\{p_{1},p_{2},\cdots ,p_{n}\right\}$ is obtained according to the stay point time sorting, and $p_{n}$ is the region label.

#### 4.2.2. Building and Prediction of Variable-Order Markov Model

**Figure 2:**
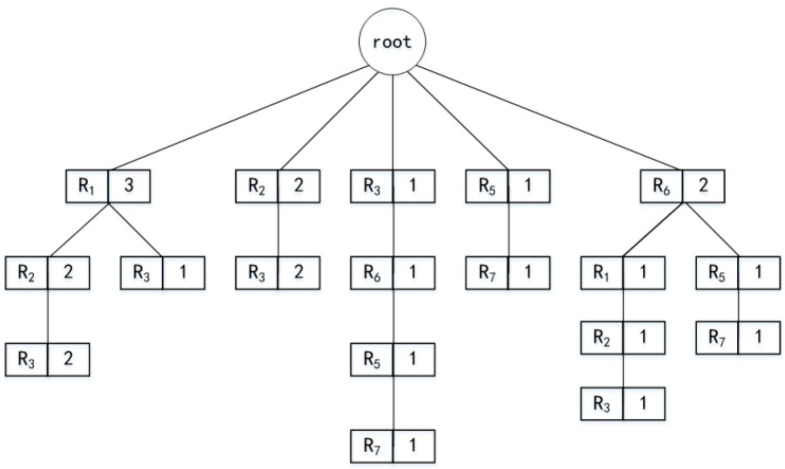
Trajectory tree generated from user history trajectories.

This paper uses a partial matching algorithm to predict the user’s next location. A maximum order *m* is set to construct a trajectory tree *T*, the maximum depth of *T* is *m* + 1. Each branch in the tree represents a user’s historical trajectory, and each child node, except the root node, consists of a region number and the number of occurrences of the trajectory. Suppose the user’s historical moving trajectories are R_3_->R_6_->R_5_->R_7_, R_1_->R_2_->R_3_, R_6_->R_1_->R_2_->R_3_, R_1_->R_3_, and according to these trajectories to construct a third-order tree, the trajectory tree is shown in Figure 2. The time complexity of generating *T* is O(*n*), and *n* is the number of training sets. When a user needs location services, we update the trajectory tree using the user’s new trajectories to ensure the trajectory tree’s availability, regardless of whether the user initiates a query to LPS. The user’s current trajectory *L_U_* is matched in depth in *T* by the find (*T*, *L_U_*) method, and if the corresponding trajectory can be matched in *T*, the current order Markov model is used for prediction, and all possible results are stored in the candidate set *Z’*. When the m-order prediction is completed, the prediction stops if the number of positions in *Z’* is greater than or equal to *k* − 1. The first *k* − 1 locations with the largest prediction probability are selected to form a *k*-anonymous set with the user’s current location. Otherwise, the location with the longest time interval from the user’s current location is removed and matched again. The newly predicted location points that do not exist in *Z’* are stored in *Z’*, and the above steps are repeated until the number of locations in *Z’* satisfies *k* − 1 or the end of the first-order model prediction.

The variable-order Markov prediction algorithm is as follows.

Because the execution time of Algorithm 1 is determined by *k* and *m*, the time complexity is max(O(*k*), O(*m*)).
**Algorithm 1:** Variable-Order Markov Model PredictionInput: *L_U_*, *T*, *k* Output: *Z′* 1: *Z′* = ∅; 2: while length (*Z′*) < *k* − 1 do 3:         if length(*L_U_*) == 0 4:             return *Z′*; 5:             break; 6:         while find (*T*, *L_U_*) return false do 7:                 *L_U_* = delete the earliest position point of *L_U_*; 8:         m = length(*L_U_*); 9:         *Z′* = use m-order Markov model to predict all possible location points; 10:       if num(*Z′*) ≥ *k* − 1 11:           return *Z′*; 12:           break; 13:       else 14:                 *L_U_* = delete the earliest position point of *L_U_*;

#### 4.2.3. Generate Anonymous Sets

**Figure 3:**
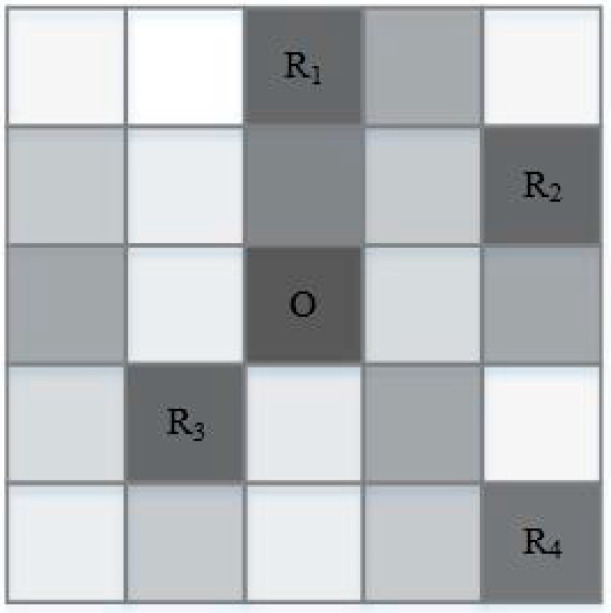
Selection POI to construct anonymous sets.

In this paper, we combined Markov prediction results *Z′* and cache contribution to construct *k*-anonymous sets and used the anonymous sets to initiate location service queries. The Markov model is used to predict the *k* − 1 most likely locations of users into *Z′*. When the Markov prediction results do not satisfy *k*-anonymity or there is a zero-frequency problem, the appropriate locations are selected by the location cache contribution to generate the anonymous set. Cache contribution refers to the degree to which the query probability of a location unit affects the cache hit rate. When the location unit data is already in the cache, adding the unit data to the cache list again has no effect on the cache hit rate. The higher the query probability of a location unit, the higher its contribution, so location points that do not exist in the cache and have a high query probability should be added to the query set. The location query probabilities are calculated based on the user’s historical query records, and the obtained probabilities are used to generate a table *S* and stored locally. The anonymous set is generated by selecting the *n* regions with the largest contribution within a certain range *R* of the user’s region. As shown in Figure 3, in the 5 × 5 size area grid, the color shade represents the size of the query probability, and O is the user location. When two location points are needed to be added to the anonymous set, it is known from the figure that R_1_, R_2_, R_3_, and R_4_ are the regions with the highest query probability. If R_1_ and R_4_ have been cached locally, then the points R_2_ and R_3_ are selected to be merged with the Markov model prediction points to generate the anonymous set.

For the generated *k*-anonymity set, the attacker can infer the user’s location based on the probability of the user initiating a service request at a location point, e.g., the attacker will exclude the location points with small query probability and some location points that should not appear at certain moments. By differential privacy protection, anonymous sets can be protected from attackers launching background knowledge attacks against them. Differential privacy protects user privacy by adding noise to the data. In this paper, by adding the Lap(*ε_i_*) of Laplace noise satisfying *ε*-difference privacy to *x_n_* and *y_n_* of each location point *l_n_*(*x_n_*, *y_n_*) in the anonymous set, respectively, the LSP receives the noise-added location points to avoid obtaining the user’s real location.

The algorithm for generating the anonymous query set is as follows.

It is easy to see that the time complexity of Algorithm 2 is the time complexity of the sorting algorithm O(*k*log*k*).
**Algorithm 2**: Anonymous Query Set Generation AlgorithmInput: *k*, *Z′*, *S*, *R*, Local cache location dataset *O*, User’s current location *loc* Output: Anonymous location set *Z* 1: *Z* = ∅; 2: if length(*Z′*) ≥ *k* − 1 3:         sort points in *Z′* by predicted probability; 4:         return *Z* = the first *k* − 1 points in *Z′* + {*loc* + Lap(*ε_i_*)}; 5: else 6:         *Z* = *Z′*; 7:         *S′* = points in *S* that are in the range *R*; 8:         sort points in *S′* by query probability; 9:         *i* = 0; 10:       while length(*Z*) < *k* − 1 do 11:                 if *S′*[*i*] is not in *Z* and *S′*[*i*] is not in *O* 12:                         *Z* + = *S′*[*i*]; 13:                         if length(*Z*) == *k* − 1 14:                                 break; 15:                 *i* + + ; 16:       return *Z = Z* + {*loc* + Lap(*ε_i_*)};

## 5. Security Analysis

The security analysis of the proposed scheme in this paper was carried out for the attack model proposed in Section 3.3.

When users need location services, there are two cases; the first is that users find the required information from the local cache, then users do not need to initiate a query to LSP, and there is no risk of location leakage. The other is that the users cannot find the data to meet the demand in the local cache, and then the users need to send a query request to LSP. We combined variable-order Markov models and the location’s cache contribution to generate *k* − 1 locations and constructed *k*-anonymity sets with the users’ current locations. In Algorithm 2, we added a privacy budget of *ε_i_*=*ε*/*k* Laplace noise to each location point in the *k*-anonymity set, and according to the serial combination property of differential privacy, Algorithm 2 satisfies $\sum_{i=1}^{k}\varepsilon_{i}$-differential privacy. By launching a query through the noisy *k*-anonymity set, the attacker has only 1/*k* probability of obtaining the noisy user’s location and cannot obtain the user’s real location.

Laplace noise is a set of random values satisfying Laplace distribution, and its basic principle is to add noise obeying Lap(*ε_i_*) to the data so that the data after adding noise satisfies the differential privacy constraint effect. The Laplace noise is added in Algorithm 2, which satisfies differential privacy. The proof procedure is as follows.

Given the generated location set is $Z=\left(l_{1},l_{2},\cdots ,l_{k}\right)$, the user’s actual location is *l*, and the generated location point after adding noise is *l_a_*(*x_a_*, *y_a_*), for any location point *l_m_*, *l_n_* in the location set, *l_a_* and *l_m_*, *l_n_* should satisfy the following relationship:

(10) $$\frac{Pr\left(x_{m}\right)}{Pr\left(x_{a}\right)}\leq e^{\varepsilon_{i}}\frac{Pr\left(x_{n}\right)}{Pr\left(x_{a}\right)}\;\frac{Pr\left(y_{m}\right)}{Pr\left(y_{a}\right)}\leq e^{\varepsilon_{i}}\frac{Pr\left(y_{n}\right)}{Pr\left(y_{a}\right)}$$

According to the probability density function of the Laplace mechanism, it is known that:

(11) $$\frac{Pr\left(x_{m}\right)}{Pr\left(x_{a}\right)}=\frac{\varepsilon_{i}}{2\Delta f}e^{-\frac{\varepsilon_{i}}{\Delta f}\left|x_{a}-x_{m}\right|}\;\frac{Pr\left(y_{m}\right)}{Pr\left(y_{a}\right)}=\frac{\varepsilon_{i}}{2\Delta f}e^{-\frac{\varepsilon_{i}}{\Delta f}\left|y_{a}-y_{m}\right|}$$

According to the trigonometric inequality, we know that,

(12) $$\left|a-m\right|\geq \left|a-n\right|-\left|m-\;n\right|$$

Therefore,

(13) $$\frac{\varepsilon_{i}}{2\Delta f}e^{-\frac{\varepsilon_{i}}{\Delta f}\left|x_{a}-x_{m}\right|}\leq \frac{\varepsilon_{i}}{2\Delta f}e^{-\frac{\varepsilon_{i}}{\Delta f}\left|x_{a}-x_{n}\right|}e^{\frac{\varepsilon_{i}}{\Delta f}\left|x_{m}-x_{n}\right|}\;\frac{\varepsilon_{i}}{2\Delta f}e^{-\frac{\varepsilon_{i}}{\Delta f}\left|y_{a}-y_{m}\right|}\leq \frac{\varepsilon_{i}}{2\Delta f}e^{-\frac{\varepsilon_{i}}{\Delta f}\left|y_{a}-y_{n}\right|}e^{\frac{\varepsilon_{i}}{\Delta f}\left|y_{m}-y_{n}\right|}$$

(14) $$\frac{Pr\left(x_{m}\right)}{Pr\left(x_{a}\right)}\leq \frac{Pr\left(x_{n}\right)}{Pr\left(x_{a}\right)}e^{\frac{\varepsilon_{i}}{\Delta f}\left|x_{m}-x_{n}\right|}\leq e^{\varepsilon_{i}}\frac{Pr\left(x_{n}\right)}{Pr\left(x_{a}\right)}\;\;\frac{Pr\left(y_{m}\right)}{Pr\left(y_{a}\right)}\leq \frac{Pr\left(y_{n}\right)}{Pr\left(y_{a}\right)}e^{\frac{\varepsilon_{i}}{\Delta f}\left|y_{m}-y_{n}\right|}\leq e^{\varepsilon_{i}}\frac{Pr\left(y_{n}\right)}{Pr\left(y_{a}\right)}$$

According to the definition of differential privacy, it can be obtained that the proposed algorithm in this paper satisfies differential privacy.

## 6. Experiment

In this section, the effectiveness and efficiency of the proposed scheme are evaluated by experimental analysis, and the cache hit rate, query time, and degree of privacy protection are compared with existing schemes.

### 6.1. Experimental Simulation Settings

The Gowalla dataset and the Geolife dataset [30] were used to verify the performance of the designed scheme. Gowalla collects user locations through their check-ins and contains 6,442,890 check-in data. Geolife contains 17,620 trajectory information generated by 182 users over five years. Both datasets contain the user’s number, time, and the corresponding latitude and longitude. Our scheme mainly considers location protection in the users’ ordinary life. In order to reduce the complexity of the trajectory tree, some trajectories outside of daily life, such as business trips and travel, were removed, and the more dense trajectories were selected as the experimental data. Ninety percent of the user trajectories were randomly selected for constructing the trajectory tree, and the remaining 10% were used as the test values. The scheme was implemented using Python 3.6 programming, the operating system was Windows 10 Home Edition, the computer CPU model was Intel i7, and the memory capacity was 64 GB. The scheme proposed in this paper was compared with LPPS [10], LPPM [11], CBPP [23], and LPADP [30], where the LPADP scheme does not use caching techniques, and the other three schemes use caching techniques. The main parameter values are shown in Table 2, and the average of 100 experiments was taken for each group of experimental results.

### 6.2. Data Availability

**Figure 4:**
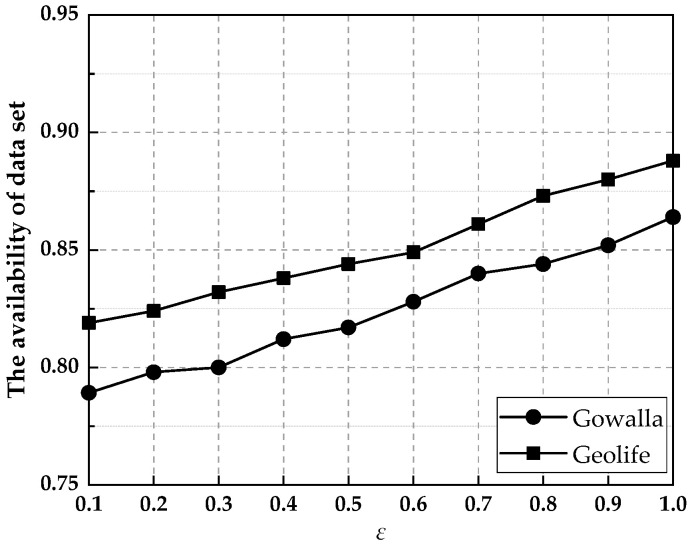
The effect of *ε* value on the availability of the datasets generated with the Gowalla and Geolife datasets.

Data availability reflects the accuracy of the anonymized data obtained from the original data after noise is added to launch the query. For the location set generation function *f*, let *f*(*Z*) be the original location set and *f*(*Z′*) be the location set after adding noise, for data availability can be defined as.

(15) $$A=1-\frac{\Delta f}{e^{\varepsilon /2}}$$

where the sensitivity $\Delta f=\underset{Z,Z^{\prime }}{\max}{\left|\left|f\left(Z\right)-f\left(Z^{\prime }\right)\right|\right|}_{1}$. In the simulation experiments, by choosing different scale parameters ($\frac{\Delta f}{\varepsilon }$), we observed the data availability of the proposed scheme under different privacy budgets *ε*. Figure 4 shows the availability of the dataset generated by this paper’s scheme under the Gowalla and Geolife datasets. The larger the privacy budget *ε*, the lower the degree of privacy protection and the higher the data availability. The proposed scheme in this paper adds Laplace noise to the positions in the anonymized set after generating the anonymized set and has high usability on both Gowalla and Geolife datasets, proving that our scheme can guarantee the usability of the query results.

### 6.3. Cache Hit Rate

**Figure 5:**
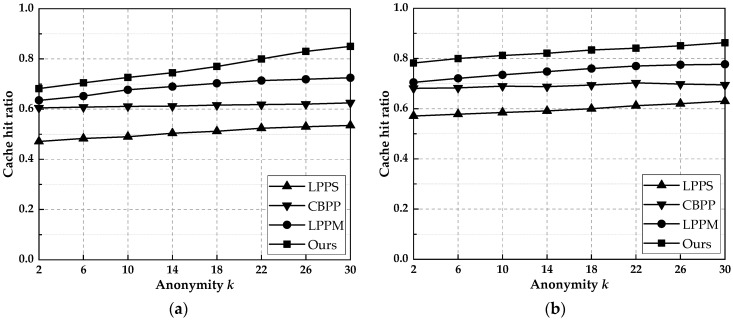
The cache hit ratio of this paper’s scheme with other schemes for different *k* values. (**a**) Gowalla. (**b**) Geolife.

The cache hit ratio reflects the performance of the caching technique in protecting the location in the proposed scheme. Figure 5 shows the cache hit ratio of this paper’s scheme with LPPS, CBPP, and LPPM schemes at different *k* values in the Gowalla and Geolife datasets. As shown in Figure 5, when the value of *k* increases, the number of locations in the set of locations generated at the time of query initiation increases, and more query results are obtained from the LSPs, which makes more cached data added to the location and updates to the data in the cache, so the cache hit rate increases with *k*. The LPPS scheme randomly generates *k* − 1 locations with real locations to form a *k*-anonymous set and uses the query results to update the cached data. However, it does not consider future user queries, and many cached query results have no relationship with users’ future queries, so the hit rate is low. The CBPP scheme obtains cache information from local and neighbors and constructs *k*-anonymity sets using *k*-diversity when the cache cannot satisfy the user, so that each location point in the anonymity set initiates a different query request. Hence, the size of the *k*-value has little effect on improving the hit rate. The LPPM scheme randomly generates virtual locations based on Markov prediction probabilities. It has a higher hit rate, but the increase in hit rate becomes smaller as the *k* value increases because the zero-frequency problem is not considered. The scheme in this paper combines Markov prediction results and cache contribution to construct anonymous sets. It solves the problem that Markov prediction results do not reach *k*-anonymity when generating anonymous sets, which have the highest cache hit rate compared to the other three schemes. In the same case, the scheme in this paper is, on average, 44.3, 21.8, and 10.5% higher than LPPS, CBPP, and LPPM, respectively.

### 6.4. Query Time

**Figure 6:**
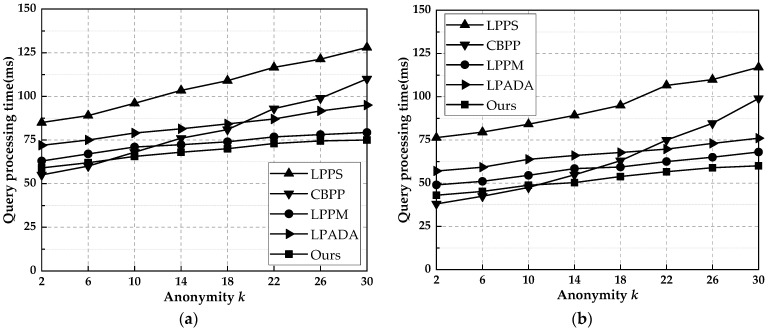
The query times of this paper’s methods and the other four methods for different *k* values. (**a**) Gowalla. (**b**) Geolife.

The query time reflects the time efficiency of the scheme from generating the anonymous set to obtaining the query results when the query needs to be initiated. Figure 6 shows the query times of the method in this paper and the other four methods for different *k* values. Among them, the LPPS scheme has the lowest hit rate and requires the largest number of queries to be launched to the LSP; therefore, the query time is the largest. The CBPP scheme has the shortest query time when the value of *k* is small and rises fastest as the value of *k* increases. Because CBPP initiates a query to its neighbors, when no query information is found in the nearby neighbors, it disperses the lookup according to the number of hops until the maximum number of hops is reached or the query result is found. LPPM, LPADA, and the scheme in this paper all use Markov prediction models. In this paper, we used a trajectory tree in the variable-order Markov model to store historical user trajectories, and the number of trajectory occurrences was marked in the trajectory tree nodes. When using the Markov model to find user trajectories, we can judge the probability of the user appearing at location points in the future based on the number of trajectory occurrences, which avoids unnecessary repeated queries, reduces the spatial complexity, and improves the finding efficiency. Compared with the other schemes, our scheme had shorter query times, which were 40.1, 11.4, 5.3, and 14.6 ms less than LPPS, CBPP, LPPM, and LPADA on average, respectively.

### 6.5. Degree of Privacy Protection

**Figure 7:**
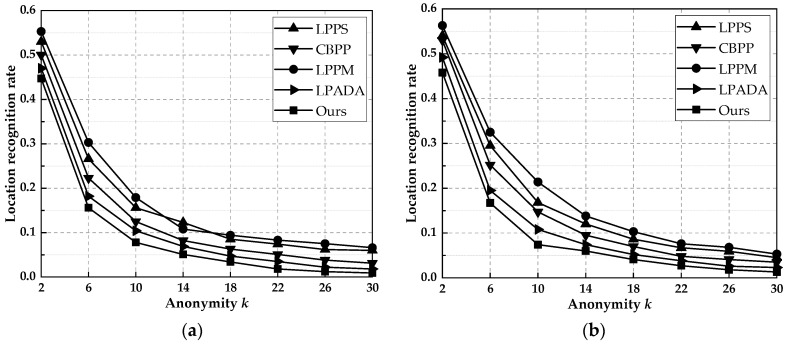
Probability of location leakage when user initiates queries with different *k* values. (**a**) Gowalla. (**b**) Geolife.

We constructed the attack model using the attack method proposed in Section 3.3. After the attacker obtains the set of locations submitted by the user, they first exclude unreasonable location points by probabilistic attacks, then narrow the range of possible locations of the user by homogeneity attacks, and finally determine the probability of location leakage using background knowledge attacks. We use the last obtained probability to evaluate the degree of location protection of the proposed scheme. As shown in Figure 7, the LPPM and LPPS schemes construct *k*-anonymity sets without using protection measures, and the probability that the actual location is discovered is larger. The CBPP scheme uses received neighbor queries to construct anonymous sets, which have higher location authenticity and can better resist background knowledge attacks. LPADA and our scheme add noise perturbation to the anonymous set, which significantly protects the user’s actual location; therefore, the security is the highest. By experimental comparison, the location recognition rate of our scheme was, on average, 39.2, 28.6, 44.6, and 10.9% lower than LPPS, CBPP, LPPM, and LPADA, respectively.

In conclusion, the above experimental results show that the proposed scheme satisfies the location privacy protection while improving the local cache’s cache hit rate and can guarantee data availability and shorter query time.

## 7. Conclusions

This paper proposes a cache-based continuous location query protection scheme to address the privacy leakage problem in continuous location queries. Users can obtain the requested location service through the local cache, which improves resource utilization. When a query needs to be launched to the LSP, a query scheme is designed to improve the cache hit rate by combining a variable-order Markov model and cache contribution degree to construct an anonymous set. We added Laplacian noise to the anonymization set to reduce the probability of leakage of the user’s location. The query results are used to update the cache information and ensure the availability of local data. By analyzing the usability and security of the proposed scheme, it was demonstrated that our scheme provided a good trade-off between user privacy and utility, and the performance was significantly improved.

Although we achieved continuous location query protection for users in this paper, the users’ personalized privacy needs have not been considered. In future work, we will work on personalized privacy protection for users, evaluate their query requests, and enhance the protection of queries with high privacy needs.

## Figures and Tables

**Table 1:** The important symbols.

Symbol	Description
*k*	Number of location points in an anonymous set
*ε*	Privacy budget
$\left(x_{i},y_{i}\right)$	Grid area identifier for the location point
*data*	Location point query data
*t*	Time of data availability
*L*	User history track
δ	Speed threshold
*T*	History trajectory tree
*m*	Maximum order of trajectory tree
*S*	Location query probability table
*R*	Anonymous point query selection range
*Z′*	Anonymous candidate set
*Z*	Anonymous location set
*O*	Local cache location dataset

**Table 2:** Parameter values in the experiment.

Parameter	Values
Number of anonymous locations *k*	2–30
Privacy budget *ε*	0.1–1.0
Time of data availability *t*	30 min
Speed threshold	0.25 m/s
Maximum order of trajectory tree *m*	3
Anonymous point query range *R*	0.5 km

## Data Availability

The experimental data used to support the findings of this study are available from the corresponding author upon request.
